# Diversity and ambivalence in general practitioners’ attitudes towards preventive health checks – a qualitative study

**DOI:** 10.1186/1471-2296-13-53

**Published:** 2012-06-08

**Authors:** Anne Søndergaard, Bo Christensen, Helle Terkildsen Maindal

**Affiliations:** 1 Department of Public Health, Section for General Practice, Aarhus University, Aarhus, Denmark

## Abstract

**Background:**

Systematic preventive health checks in primary care have been introduced in several countries. The Danish Health Service does not provide this service, but health checks are nevertheless being conducted unsystematically. Very little is known about the GPs’ experience with this service.

The purpose of our study is to describe GPs’ attitudes towards and concerns about providing preventive health checks and to describe their experiences with the health checks that they provide in daily practice.

**Methods:**

A qualitative descriptive study was conducted based on three semi-structured focus group interviews with 16 GPs from Central Region, Denmark. The focus group interviews took place at the Department of Public Health, Section for General Practice, Aarhus University in November 2010.

**Results:**

We found that the participating GPs all conducted some kind of preventive health checks, but also that there was great diversity in the content. The GPs were somewhat ambivalent towards health checks. Many GPs found the service beneficial for the patients. Concurrently, they had reservations about promoting ill-health, they questioned whether the health checks were a core mission of primary care, and they were concerned whether the health checks would benefit the “right” patients. The GPs felt a need for further documentation of the benefits for the patients before a possible future implementation of systematic health checks. Some GPs found that health checks could be performed in other settings than general practice.

**Conclusions:**

Our study revealed that health checks are performed differently. Their quality differs, and the GPs perform the health check based on their personal attitude towards this service and prevention in general. Our analysis suggests that the doctors are basically uncertain about the best approach. Our study also uncovers the GPs’ reservations about inducing negative psychological reactions and decreased well-being among the health check participants. Further studies are needed to disclose where these concerns emerge.

## Background

Preventive health checks have been implemented systematically in primary care in several countries [1,2]. In Denmark, the possibility of implementing a similar service through general practice has been discussed for several years [3,4]. In 2009, the Danish Preventive Commission stated that a targeted screening for cardiovascular diseases through general practice may have a positive effect on health, but that the evidence was sparse [5].

In Denmark, no systematic programme for preventive health check exists, but from 2006–2010, the collective agreement for GPs included the possibility of conducting health checks that focused on the systematic prevention of prevalent diseases, such as cardiovascular disease and diabetes. GPs receive a preventive consultation fee for performing health checks. This agreement was changed in 2010, but still allowed the Danish GPs to carry out preventive health checks, although without receiving any special fee.

The evidence on health checks is sparse, but a small Danish study “the Ebeltoft Health Promotion Study” demonstrated that offering preventive health checks in general practice had a positive effect on cardiovascular health after 5 years with no increase to costs [6,7]. The health checks in this study were very comprehensive and included all males and females between the ages of 30–49 years.

At present, GPs can provide random health checks, but no guidelines exist about the appropriate age group, gender, general examination, blood tests etc.

The lack of guidelines leads to GPs generally making individual decisions about which patients to offer health checks to and what the health check should include. The authors’ pre-understanding was that Danish GPs are experienced with preventive health checks. Anecdotal evidence suggests, that the GP’s are concerned about whether or not to provide this service, and to whom. Our study will contribute considerably to the clarification of the current content, target group and benefits of health checks as they are provided by GP’s today, given the lack of best practice guidelines, as our knowledge in this area is limited.

The purpose of our study is to describe GPs’ experiences of the health checks that they provide in daily practice and their attitudes towards and concerns about providing preventive health checks.

## Methods

This qualitative descriptive study was based on data from three semi-structured focus group interviews with 16 GPs from Jutland, Denmark (Central Region). The qualitative descriptive design was chosen because we wanted to stay close to the data obtained and describe the informants’ points of view in a language similar to their own. Qualitative description is a method that affords a comprehensive summary of experiences without an in-depth level of interpretation [8]. We enhanced rigour by paying attention to the voices of the informants (authenticity), staying true to the topics under investigation (credibility), being reflective and critical to both the results and the decision made throughout the research process (criticality) and by having an ongoing reflection and self-criticality of the researcher (integrity) as recommended by Milne and Oberlee [9].

### Informants and recruitment

The informants were Danish GPs recruited through the Danish database of general practices. This database stores information on all Danish GP including age, gender, practice localisation, number of patients, and whether nurses are employed or not. The GPs were purposefully selected for strategic variation in age, gender, practice location and variation in the use of the preventive consultation fee.

We contacted 100 GPs by letter and subsequently by telephone. Twenty-three agreed, 16 of whom participated, Seven either failed to attend or sent their apologies. The main reason given for not participating was lack of time. The participants mean age was 53 years. One third were women, one third were located in Aarhus (approx. 500.000 citiziens), one third in villages, in the countryside and the rest in medium size cities and suburbs.

### Interviews

Three focus group interviews with three, five and eight participants, respectively, were conducted by AS and were supervised by either HTM or BC. After the third focus group interview, we had accomplished saturation on the themes we wanted to explore. No new perspectives appeared, and we therefore decided not to invite any more GP’s.

The focus group interviews took place at the Department of Public Health, Section for General Practice, Aarhus University in November 2010. The focus group interviews were conducted using a topic guide [10] with a design based on clinical experience, extensive literature studies on preventive health checks and discussions among the authors. Co-authors HTM and BC have both done research in prevention in general practice for several years.

The themes of the topic guide were: Do GPs consider health checks as a task for general practice? Do the GPs have any barriers in connection with performing health checks? How can we best organise health checks? Which clinical examinations and laboratory tests do GPs find relevant in a health check? (See Additional file 1: Appendix 1 for further details).

The topic guide had the same themes in the three focus groups interviews, but our pre-understanding and perspectives changed during the data collection. Although the questions in the topic guide were unchanged, the questions were arranged in a different sequence. The focus group interviews began with an introduction of the interviewers and participants as well as information about anonymity and publication, which was accepted. Information about the study aim was provided and informed consent obtained. At the end of each group interview, the interviewer presented some tentatively identified issues to the participants for clarification or confirmation.

### Analysis

The focus group interviews were recorded and subsequently transcribed verbatim by AS. All transcripts were read repeatedly to ensure familiarity with the material before coding. Meaningful text units were identified and coded by AS, the text units were grouped into relevant categories and the main codes were identified, and subsequently grouped into three main shared categories.

The authors reached agreement on coding and categorisation after careful discussion. We ensured a valid analysis by having an on-going discussion about the results in the group of authors.

### Ethics

Ethical considerations: In accordance with Danish law, it was not necessary to notify any authority about the study. The principles of good research ethical practice were followed [11]. The GPs were informed about the study by letter and telephone. They were free to decide, whether they would participate in the focus group or not. No reminders were sent, nor was any further contact made, if they did not respond to the invitation. At the start-up of each focus group, the aim and conditions were clarified and informed consent was achieved. The participating GPs were paid for two hours of work according to the collective agreement for GPs.

## Results

Thirteen main codes were identified and grouped into three main categories: 1) Diversities in the delivery of health checks, 2) the GPs’ ambivalence towards health checks, and 3) the GPs’ request for clarification about health checks (See Additional file 2: Appendix 2).

### Diversity in the delivery of health checks

All the GPs conducted some kind of health check in their daily practice.

There was a relatively large difference in the number of health checks conducted on a monthly basis, which varied from one to twenty per doctor. The doctors suspected that this variation was due to various reasons, amongst others the different patient populations or whether the patients experienced the health check as a service on equal terms with, e.g., preventive health care for children.

We found four different approaches to health checks: A) a standard set-up including medical history, a fixed set of blood tests and a physical examination, sometimes with an electrocardiogram and spirometry, B) a procedure depending on the patients’ individual expectations towards a health check, C) a procedure depending on medical history, and D) no regular procedure, but the content of the examination was decided from time to time and corroborated with the patient’s condition.

The GPs reported that it was also important to focus on mental health. This often had low priority in the existing health checks. More informants agreed that of the few, single questions asked during the check-up, one could be about mental health.

"“It doesn’t really have to be a lot of questions (about mental health). How are you, your work, your relationship with your wife or whatever, right?” Informant 10."

A typical reason for requesting a health check was the patients’ health worries caused by concern for and due to the ill health of relatives or friends. Another reason could be their participation in municipal health or community-based programmes. The programme staff would recommend the participants see their GP if, for example, their cholesterol level was elevated. A third reason for requesting a health check could be that the patients felt obliged towards family members or other people in their network. They felt, that staying healthy was their responsibility towards family and friends.

"“It comes in waves, but a lot of them come because they have had a health check at their workplace or at the chemist’s or wherever. Then they are told that their blood pressure is too high and that they should consult their GP. And suddenly I have a crowd of people wanting everything checked!” Informant 7."

Some GPs stressed the importance of giving informed consent regarding health checks. The patients’ expectations towards the examinations became more realistic, and it gave the GPs the option to motivate them for lifestyle changes or prescribe medication if necessary.

"“I usually ask them what they understand by a health check and what they want it for.” Informant 3."

### The GPs’ ambivalence towards health checks

A recurring topic was whether health checks were considered a core mission in primary health care. Several informants considered prevention to be an important part of their work, while others viewed health checks as peripheral to their field of work. Some GPs expressed the opinion that health checks were more of an educational task than a service provided by a GP.

"“We are fed up maintaining cholesterol levels. We want to do something else. Retired schoolteachers could do that job. It is more an educational thing than a doctor’s.” Informant 13."

Although some of the GPs did not regard health checks as a core task, all the participants did perform some kind of health check. None of them, even those who were against health checks, would refuse a patient who requested a health check.

"“I have to say, that I never say no. What I use as an excuse is that the Danes are constantly being barraged with good advice from neighbours, colleagues and the media. And it must be difficult for laymen to keep updated.”, Informant 16."

The fact that the patients see themselves as consumers is another reason why the doctors perform health checks. Several doctors reported that the patients demanded that they did the health check. The patients regarded it as their right and viewed health checks as a service just like any other consultation.

"“Patients nowadays see themselves as consumers. And modern consumers do not want to hear a host of reasons why they can’t buy something, they just want the product.” Informant 4."

There was general consensus that the GPs rarely reach the “right” patients with the health check in its current form, where the patients request it themselves. Typically, the health conscientious patient attends, and the patient who would really benefit from a health check stays away.

"“But right now, there is a mismatch. Only the most conscientious show up. And the ones who need it the most don’t come. Alcoholics don’t ask for a check-up, do they?” Informant 11."

Despite the ambivalence, several doctors believed that in many ways the health checks did do some good.

"“People, who would not otherwise have come, show up. I think it is worth it, at any rate among my patients who are predominantly elderly people and people from the lower social classes.” Informant 7."

The risk of inducing negative psychological reactions and false security by performing health checks worried several of the GPs.

"“But honestly, it makes my flesh crawl when you talk about health checks for everyone… The risk is that we are checking up on the wrong patients and causing a delay because our patients then think that everything is wonderful, and that it is - well - the wrong target group”, Informant 3."

"“Concerning these young people, I think that it (the health checks) does them more harm than good. My personal view is, talk about health, but don’t measure their cholesterol level.” Informant 10."

### The GPs’ request for clarification

The third main theme was the doctors’ need for clarification on the efficacy and organisation of preventive health checks before being able to take a stand on whether it would be beneficial to provide the Danish population with systematic health checks.

"“… I haven’t seen any documentation. Because if somebody tells me that health checks are really beneficial, then I will do them, - correction, my nurse will do them!” Informant 14."

There was general concern about the organisation of systematic health checks and whether they were too comprehensive to be manageable in general practice. If the number of examinations increases, what can GPs accomplish in everyday practice?

"“I think we can do it, we have the qualifications - the question is whether we have the time for it.” Informant 5."

On the basis of the above-mentioned reasons, some GPs were of the opinion that the health checks could be carried out in other settings than general practice, e.g., at the workplace or in a municipal setting. However, some expressed concern as to whether the quality of the examination would be satisfactory.

"“Is it necessary to do this in general practice? Or should we just keep it quietly to ourselves? I’m not afraid of promoting general screening, but I think that the more targeted screening procedures are under our jurisdiction. So I would like to see it done in the workplaces, providing it is performed by qualified people.”, Informant 8."

## Discussion

### Summary of main findings

We found that the participating GPs all conducted some kind of preventive health check, although the content of the health checks varied greatly. The study revealed a considerable ambivalence in the GPs’ attitudes towards health checks. The GPs would never deny a request for a health check, but they were not sure that the service was offered to the right patients. Our study also uncovers the GPs’ reservations about inducing negative psychological reaction and decreased well-being among the health check participants. The GPs demanded evidence of efficacy and organisation of preventive health checks.

### Comparison with existing literature

The doctors performed health checks to a greater or lesser extent, and the service differed from practice to practice. The fact that the participants in our study actually performed health checks was expected, as they volunteered to the focus group interviews. In a recent Dutch questionnaire study with 330 GPs, one quarter of the respondents reported that they actively invited patients to preventive checks [12]. Our study design cannot reveal an exact estimate of the dissemination in Denmark, but the reality of the checks being performed differently is obvious.

This was in accordance with our expectations, as there are no available Danish guidelines for health checks [13]. This great diversity is similar to the findings of a recent Australian study; GPs’ preventive behaviour concerning lifestyle behavioural risk factor screening and management factors varied and was influenced by personal interest, perceptions of their own effectiveness and perceived external control factors [14]. In general, the doctors agreed that the patients should give informed consent before the health check was performed. Informed consent regarding screening for cardiovascular disease in general practice has gained approval in, amongst other places, the United Kingdom. The reported advantages include achieving a more effective intervention among people who choose to participate, and these screenings are consequently more cost-effective than traditional screening programmes [15].

Participating doctors in this study reflected a variety of different views regarding health checks, not previously identified in the literature. Some GPs seemed to be afraid of inducing negative psychological reactions and decreasing well-being in the population –a concern not supported by other Danish studies in general practice, which report that health checks do not impair mental well-being [16-18]. At the same time some participants were concerned that if they did not do the health checks, other professions or sectors would, whilst other participants felt that health checks were an educational task which did not require the input of doctors. Such differences in doctors’ perceptions of health checks reflects the lack of clear evidence and guidelines for undertaking health checks.

Nevertheless, none of the doctors we interviewed would ever reject a patient who wanted a health check despite the fact that they did not see it as a core task. The stance of not seeing it as a core task is comparable with the results of an Australian study about doctors’ attitudes towards health checks [2]. This underlines that health checks are not ranked alongside general prevention, as many of the doctors saw prevention as a natural part of their work, in line with Danish studies [19].

Many of the doctors we interviewed found that they did not always reach those patients that needed the health check the most. A study dealing with screening for cardiovascular disease showed that non-participants more often come from deprived social groups, have cardiovascular diseases or are genetically disposed to it and are smokers [20]. Some doctors, however, thought it was of great use. These internal disparities may express a considerable difference in the doctors’ patient populations regarding geography, mean age and social classes.

A Dutch study on the prevention of cardio-metabolic diseases in general practice found that GPs had a positive attitude towards primary prevention, but also that the primary prevention should focus on patients at risk, e.g., family history, obesity and smoking [12]. The challenge of finding the right risk factors was expressed by the Danish GPs in this study, originating in the on-going theme of being concerned about reaching the patients at “real” risk.

### Strengths and limitations of the study

We chose a qualitative approach owing to the sparse literature about doctors’ attitudes towards and experiences with preventive health checks. Our results are based on focus group interviews with a small group of GPs with different backgrounds and professional experience. 16 of 100 invited participated. We assumed that those who volunteered to the focus group would have a special interest in health checks and thereby represent the diversity among GPs. This assumption was confirmed during the interviews.

The strength of focus group interviews is that interaction between the focus group members can contribute to exploring and clarifying individual opinions and enhance the breadth of data through mutual reflection. Concurrently, the interaction in the group can introduce new issues to the interview, enabling new themes or aspects to materialise [21]. A number of new aspects and issues on the different subjects emerged from our focus group interviews. However, commenting on these issues would surpass the aim of our study and analysis.

The flexible topic guide helped to promote authenticity by giving all the informants the freedom to speak as fully as possible and to present their perceptions as accurately as possible.

Methodological problems may arise when doctors interview doctors and question credibility. As part of a group, one can benefit from being part of a mutual culture. On the other hand, the informants’ answers may be influenced by a wish to project a positive professional identity. We were not under the impression that the doctors wanted to please us, and the identified four different approaches towards health checks also contradict this and reflect the on-going national debate about health checks [3,22].

The dual role of AS as both interviewer and researcher represents a potential source of bias. On-going reflection and awareness about this duality was crucial throughout the study. We also tried to promote participant-driven data by making sure that throughout the analysis the categories emerged from the data, and not from the subheadings in the topic guide. We believe that the above-mentioned approach was critical to the integrity and criticality of the study.

## Conclusions

Our study revealed that there is considerable variation in the content and quality in the health checks undertaken by Danish GP’s, who perform the health check based on their personal attitude towards this service and prevention in general. Our analysis suggests that the doctors are basically uncertain about the best approach. A best practice guideline for health checks would therefore be very advantageous. Accordingly, implementation of clinical guidelines must be followed by discussions about health promotion attitudes if a change in routine GP practice is sought. Our study also uncovered the GPs’ reservations about inducing negative psychological reactions and decreased well-being among the health check participants. Further studies are needed to disclose where these concerns emerge.

## Competing interests

The author(s) declare that they have no competing interests.

## Authors’ contributions

AS conducted the focus group interviews and transcribed them. AS also did the primary coding of text units. BC and HTM supervised the focus group interviews. The analysis was conducted by AS, BC and HTM. All authors read and approved the final manuscript.

## Funding

Multipraksisudvalget paid the participating doctors.

## Pre-publication history

The pre-publication history for this paper can be accessed here:

http://www.biomedcentral.com/1471-2296/13/53/prepub

## Supplementary Material

Additional file 1 **Appendix 1.** Topic guide – word document. The topic guide used in the focus group interviews. Click here for file

Additional file 2 **Appendix 2.** Main categories and codes – word document. The table of codes and main categories to explain how codes transformed into main categories in the analysis. Click here for file
