# Supplementary material for: Diversity and ambivalence in general practitioners’ attitudes towards preventive health checks – a qualitative study
Source: BMC Fam Pract. 2012 Jun 8;13:53. doi: 10.1186/1471-2296-13-53 (PMC3406979; doi:10.1186/1471-2296-13-53)
Supplement: Additional file 1 — Appendix 1. Topic guide – word document. The topic guide used in the focus group interviews. [file 1471-2296-13-53-S1.doc]

| Themes | Questions |
| --- | --- |
| Experience with and attitude towards health checks.  Including organisation  How do you value the health check in respect to the core mission of general practice. Importance and possible positive effects.  Barriers  Clinical and paraclinical examinations  Ending | We know that patients sometimes request health checks. What do you do when a patient asks for a health check?  How often do your patients ask for health checks?  Do you have a standard procedure for health checks – do you for example offer the same health check to a sixty-year old man and a forty-year old woman?  How much do you involve your staff in the health checks – do they take the blood tests, convey the test results or something else?  What is your fee for the health check? 0106(preventive consultation fee)?  Do you provide patient information on health checks?  Do you think Denmark should implement systematic health  checks?  Is it a task for primary care – why/why not?  How do your patients benefit from health checks?  Is it a certain type of patient who requests a health check. Are they the “right” patients – or more often than not the ones who want a pat on the back?  Who should provide health checks?  Have you ever offered a health check to a patient, during a consultation for another purpose?  And if so, how do you feel about using a proactive approach?  Does it affect the doctor-patient relationship – positively or negatively?  How do patients react to unexpected adverse results?  Do health checks boost ill health, and if so, how do you avoid this?  It has been proposed that informed consent should be given before a health check. The reason being to develop more realistic expectations towards health checks, to make patients more motivated for lifestyle changes if necessary, and promote less ill health. What is your view on this?  Should there be a specific fee for health checks?  How many working hours would you expect it to take, if systematic health checks were implemented in your practice?  If health checks were implemented every five years for everyone between 40-74 years, it would give 8 health checks per week in a practice with 1300 patients. Would this suit the way your practice is organised?  In Great Britain, health checks have been implemented for everyone between 40-74 years without existing cardiovascular disease or diabetes. The examination takes place every five years and includes a history of age, gender, ethnicity, smoking status, predispositions, physical activity, and medicine. The tests include blood pressure, height, weight, cholesterol and glucose. The physical tests are performed by a nurse or a pharmacist.  What are your views on the above?  In the above, the primary focus is on cardiovascular disease. Are there any other tests you would like to include, or should some be excluded?(screening for depression, D-vitamin status, colorectal cancer screening?)  How do you feel about filling out questionnaires, e.g. the taking the history before a health check?  How long should a health check take?  Should the content of the health check vary for different age groups?  There is an ongoing debate about health checks, do you have any comments to make?  Do you have any comments on health checks in general?  Questions?  Which of the above issues do you find particularly important?  Thank you for participating! |
