# Supplementary material for: Diversity and ambivalence in general practitioners’ attitudes towards preventive health checks – a qualitative study
Source: BMC Fam Pract. 2012 Jun 8;13:53. doi: 10.1186/1471-2296-13-53 (PMC3406979; doi:10.1186/1471-2296-13-53)
Supplement: Additional file 2 — Appendix 2. Main categories and codes – word document. The table of codes and main categories to explain how codes transformed into main categories in the analysis. [file 1471-2296-13-53-S2.doc]

**Main categories** **Codes**

Diversities in the delivery of health checks Content of a health check

The psychological aspect

Reasons for encounter

Informed consent

The GP’s ambivalence towards health checks GP’s core task

The ”wrong” patients

The patient as consumer

Inducing negative psychological reactions

Health checks despite resistance

Utility

The GP’s request for clarification Do we need health checks?

Organisation

Evidence?
